# Supplementary material for: Mechanisms governing the pioneering and redistribution capabilities of the non-classical pioneer PU.1
Source: Nat Commun. 2020 Jan 21;11:402. doi: 10.1038/s41467-019-13960-2 (PMC6972792; doi:10.1038/s41467-019-13960-2)
Supplement: Supplementary file 7 — Source data [file 41467_2019_13960_MOESM7_ESM.zip › Source_Data/Figure6/Figure6B_MotifScanOutput/ETS1_PU.1/homerResults/motif4.similar.html]

motif4

## Information for motif4

A
C
T
G
C
G
A
T
C
G
T
A
T
C
G
A
A
G
T
C
A
G
T
C
C
T
G
A
A
T
G
C
C
G
T
A
  
Reverse Opposite:  

G
C
A
T
A
T
C
G
G
A
C
T
C
T
A
G
A
C
T
G
A
G
C
T
G
C
A
T
G
C
T
A
T
G
A
C
  

|  |  |
| --- | --- |
| p-value: | 1e-743 |
| log p-value: | -1.712e+03 |
| Information Content per bp: | 1.652 |
| Number of Target Sequences with motif | 5245.0 |
| Percentage of Target Sequences with motif | 33.13% |
| Number of Background Sequences with motif | 3416.4 |
| Percentage of Background Sequences with motif | 10.65% |
| Average Position of motif in Targets | 100.9 +/- 51.2bp |
| Average Position of motif in Background | 99.1 +/- 62.1bp |
| Strand Bias (log2 ratio + to - strand density) | -0.1 |
| Multiplicity (# of sites on avg that occur together) | 1.04 |
| Motif File: | file (matrix) reverse opposite |

### Similar de novo motifs found

|  |  |  |  |  |  |  |  |
| --- | --- | --- | --- | --- | --- | --- | --- |
| Rank | Match Score | Redundant Motif | P-value | log P-value | % of Targets | % of Background | Motif file |
| 1 | 0.858 | G A C T A C G T A C T G A G C T A C T G A C T G A C G T | 1e-523 | -1204.593297 | 46.97% | 24.51% | motif file (matrix) |
| 2 | 0.862 | G C T A T C G A T G C A A G T C A G T C G C T A A T G C G T C A T C A G C T G A T C A G G T C A G T A C T G A C | 1e-382 | -880.893393 | 18.69% | 5.96% | motif file (matrix) |
| 3 | 0.767 | G A C T A G T C C G A T A C T G A G T C A C T G A C T G A G C T | 1e-246 | -567.659970 | 21.70% | 10.10% | motif file (matrix) |
| 4 | 0.809 | C T A G A G T C A C T G C T A G C G A T C G T A C T G A | 1e-237 | -546.804138 | 17.06% | 7.01% | motif file (matrix) |
| 5 | 0.817 | T A G C G C A T A T C G T C G A T A G C G A T C G C T A T A G C G T C A T C G A C T G A A C T G | 1e-154 | -356.032018 | 8.86% | 3.05% | motif file (matrix) |
| 6 | 0.828 | A G C T A C T G A G T C A C T G A T C G A C G T T A G C C T G A C T A G C T G A | 1e-124 | -285.764737 | 9.71% | 4.08% | motif file (matrix) |
